# Interplay of Cellular mRNA, miRNA and Viral miRNA during Infection of a Cell

**DOI:** 10.3390/ijms24010122

**Published:** 2022-12-21

**Authors:** Vladimir P. Zhdanov

**Affiliations:** Boreskov Institute of Catalysis, Russian Academy of Sciences, Novosibirsk 630090, Russia; zhdanov@catalysis.ru

**Keywords:** intracellular viral kinetics, gene expression, mRNA and miRNA, mean-field kinetic equations, Monte Carlo simulations

## Abstract

The understanding of the kinetics of gene expression in cells infected by viruses is currently limited. As a rule, the corresponding models do not take viral microRNAs (miRNAs) into account. Such RNAs are, however, operative during the replication of some viruses, including, e.g., herpesvirus. To clarify the kinetics of this category (with emphasis on the information available for herpesvirus), I introduce a generic model describing the transient interplay of cellular mRNA, protein, miRNA and viral miRNA. In the absence of viral miRNA, the cellular miRNA is considered to suppress the populations of mRNA and protein due to association with mRNA and subsequent degradation. During infection, the viral miRNA suppresses the population of cellular miRNA and via this pathway makes the mRNA and protein populations larger. This effect becomes appreciable with the progress of intracellular viral replication. Using biologically reasonable parameters, I investigate the corresponding mean-field kinetics and show the scale of the effect of viral miRNAs on cellular miRNA and mRNA. The scale of fluctuations of the populations of these species is illustrated as well by employing Monte Carlo simulations.

## 1. Introduction

Kinetic models are widely used in order to illustrate and clarify various aspects of gene expression in intact cells (reviewed in [1,2,3,4,5,6]; see also recent articles [7,8,9,10,11] and references therein) and during their infection by viruses (reviewed in [12,13,14]; see also [15,16,17,18,19,20,21,22,23,24,25,26,27,28] and references therein). The models of the former category are usually focused on proteins, mRNAs, and non-coding RNAs (ncRNAs), e.g., miRNAs. The models of the latter category typically operate with the populations of cellular proteins and mRNAs; and viral proteins, RNAs, or DNAs; and virions. Now, there is, however, evidence that various host miRNAs are involved in a cellular antiviral response and that active miRNAs can also be encoded by a viral genome and expressed in the host [29]. For COVID-19, for example, the corresponding literature is reviewed in [30,31]. Overall, the intracellular pathways, including host and viral miRNAs, are complex and not yet fully understood. The corresponding kinetic models with various feedbacks have in fact not been explored theoretically and obviously merit attention.

In this context, I can note that the interplay of viral miRNA and host mRNA and protein (P) was analyzed earlier theoretically [27] by using a four-variable kinetic mean-field model for the populations of these species, with the following mechanistic steps:

$$\begin{array}{c} \mathrm{Gene}\to \mathrm{Gene}+\mathrm{mRNA},\\ \mathrm{mRNA}\to \mathrm{mRNA}+P,\\ G\to G+\mathrm{miRNA},\\ \mathrm{mRNA}+\mathrm{miRNA}\to Ø,\\ \mathrm{mRNA}\to Ø,\;\;P\to Ø,\;\;\mathrm{and}\;\;\mathrm{miRNA}\to Ø, \end{array}$$

where Gene and G represent the host and viral genomes. The cellular ncRNA was, however, not taken there into account.

Herein, I present a more complete generic kinetic model describing the interplay of viral miRNAs and cellular mRNAs, miRNAs, and proteins. To validate this work, I can refer to the replication of herpesvirus, the formation of the corresponding viral miRNAs, and their interactions with cellular ncRNAs (see, e.g., experiments [32,33,34]); regarding antiviral miRNAs, see [35]). The cellular ncRNAs can influence the population of cellular mRNAs, and accordingly the population of the corresponding cellular proteins can be influenced as well.

As a general remark for introducing the math, I recall that in kinetic models of gene expression in cells, the formation and degradation of proteins, mRNAs, and ncRNAs are usually viewed as the key elementary steps [2,3,4,5,6]. In reality, these steps occur via numerous substeps [1]. In other words, the steps under consideration are almost always coarse-grained—i.e., a single step represents a few substeps—and each substep often represents in turn a few substeps of a lower level. The extent of detalization of steps and substeps depends on the goals of an analysis. Usually, a theoretical treatment has a few goals, and accordingly, the extent of detalization can be different even in one study. With this reservation, I first introduce below a set of steps in order to keep the links with numerous earlier theoretical models [2,3,4,5,6] and a relevant experiment [34] and then simplify them in order to keep the math compact and suitable for general readership (Section 2). The results of the corresponding mean-field calculations and Monte Carlo (MC) simulations are given afterwards (Section 3) and followed by the conclusion (Section 4).

## 2. Methods

### 2.1. General Scheme

For genes of one type (as in the model under consideration), the steps resulting in the mRNA and protein formation are usually schematically presented as [2,3,4,5,6]

(1) $${\mathrm{Gene}}_{1}\to {\mathrm{Gene}}_{1}+{\mathrm{mRNA}}_{1},$$

(2) $${\mathrm{mRNA}}_{1}\to {\mathrm{mRNA}}_{1}+P_{1},$$

(3) $${\mathrm{mRNA}}_{1}\to Ø,\;\;\mathrm{and}\;\;P_{1}\to Ø.$$

Genes can be transcribed also into ncRNAs, or more specifically (in the context under consideration), into miRNAs, which are short compared to mRNA [6,36]. Mechanistically, it occurs via the formation of precursors (long premiRNAs); i.e., the corresponding steps can be represented as

(4) $${\mathrm{Gene}}_{2}\to {\mathrm{Gene}}_{2}+{\mathrm{premiRNA}}_{2},$$

(5) $${\mathrm{premiRNA}}_{2}\to {\mathrm{miRNA}}_{2},$$

(6) $${\mathrm{premiRNA}}_{2}\to Ø,\;\;\mathrm{and}\;\;{\mathrm{miRNA}}_{2}\to Ø.$$

Comparing steps (1) and steps (4) and (5), one can notice that the former step does not include pre-mRNA, whereas the latter steps contain pre-miRNA${}_{2}$, and this might appear inconsistent because in reality the formation of mRNA includes pre-mRNA as well (see, e.g., Figure 1 in [1]). As in many other kinetic models of gene expression [2,3,4,5,6], I do not introduce the substeps including pre-mRNA because they are not coupled with other substeps and accordingly are not important in the context under consideration.

The conventional scheme of the interaction of mRNA and miRNA includes their reversible association and degradation [6,37],

(7) $${\mathrm{mRNA}}_{1}+{\mathrm{miRNA}}_{2}⇌{\mathrm{mRNA}}_{1}\cdot {\mathrm{miRNA}}_{2},$$

(8) $$\begin{array}{c} {\mathrm{mRNA}}_{1}\cdot {\mathrm{miRNA}}_{2}\to {\mathrm{miRNA}}_{2}+Ø,\;\;\mathrm{and}\\ {\mathrm{mRNA}}_{1}\cdot {\mathrm{miRNA}}_{2}\to Ø. \end{array}$$

As is usual in molecular biology, these steps resemble those of the classical enzyme kinetics (with miRNA${}_{2}$ being the enzyme and mRNA${}_{1}$ the substrate to be degraded).

After infection of a cell, virions use the cellular machinery for the formation of various species needed for their replication. The corresponding steps are numerous. In the context under consideration, the attention can be focused on the viral genome transcription into viral pre-miRNA with subsequent conversion to viral miRNA (as, e.g., in the case of herpesvirus [34]),

(9) $$G\to G+{\mathrm{premiRNA}}_{3}\;\;\mathrm{and}\;\;{\mathrm{premiRNA}}_{3}\to {\mathrm{miRNA}}_{3},$$

association of viral miRNA with cellular pre-miRNA,

(10) $${\mathrm{premiRNA}}_{2}+{\mathrm{miRNA}}_{3}⇌{\mathrm{premiRNA}}_{2}\cdot {\mathrm{miRNA}}_{3},$$

and degradation of these species,

(11) $$\begin{array}{c} {\mathrm{premiRNA}}_{3}\to Ø,\\ {\mathrm{miRNA}}_{3}\to Ø,\\ {\mathrm{premiRNA}}_{2}\cdot {\mathrm{miRNA}}_{3}\to {\mathrm{miRNA}}_{3}+Ø,\;\;\mathrm{and}\\ {\mathrm{premiRNA}}_{2}\cdot {\mathrm{miRNA}}_{3}\to Ø. \end{array}$$

In addition, there are viral genome replication and degradation:

(12) G→2GandG→Ø.

The G replication is here represented as one coarse-grained lumped step. The G loss is shown as one lumped step as well. The latter step is assumed to describe all the channels of the loss, including the G degradation and the formation of premature and mature virions and their departure from a cell.

The mechanistic steps above do not contain the cellular DNA replication, and in the context of the analysis presented, this means that all the processes occur in a cell during one cell cycle. The focus on this situation is biologically reasonable and can be validated comparing the timescales of various processes occurring in human cells. In particular, the timescales of the formation and degradation of proteins, mRNA, and miRNA are typically shorter or comparable with one hour (see, e.g., Section 3.6 in [6] or [38]). The timescale of the viral genome replication is usually a few hours (see, e.g., Figure 2 for HIV-1 and IVA viruses in [21]). In contrast, the length of the cycle of human fast-dividing cells grown outside the body under optimal conditions is typically longer (approximately 24 h [38]), and the lengths of the cycles of many other cells which are relevant for infections are often much longer both in vivo and in vitro (see, e.g., the data for transformed human hepatocyte growth [39]). Taken together, this information means that the number of viral genomes formed in a cell during the cell cycle is often large, and accordingly, it makes sense to focus on this case.

### 2.2. Coarse-Grained Steps

The kinetic equations corresponding to the steps introduced above are somewhat cumbersome. To simplify the treatment, I keep steps (1)–(3) for the formation and degradation of mRNA${}_{1}$ and P${}_{1}$, steps for the miRNA${}_{2}$ and miRNA${}_{3}$ degradation (in (6) and (11)), and using one of the common approximations in the models of gene expression, replace steps (4), (5), and (9) for the miRNA${}_{2}$ and miRNA${}_{3}$ formation with two lumped steps,

(13) $${\mathrm{Gene}}_{2}\to {\mathrm{Gene}}_{2}+{\mathrm{miRNA}}_{2},$$

(14) $$G\to G+{\mathrm{miRNA}}_{3}.$$

Along this line, I replace the ${\mathrm{mRNA}}_{1}$-${\mathrm{miRNA}}_{2}$ association step (7) and the corresponding degradation steps (8) by one lumped step

(15) $${\mathrm{mRNA}}_{1}+{\mathrm{miRNA}}_{2}\to Ø.$$

By analogy, the ${\mathrm{premiRNA}}_{2}$-${\mathrm{miRNA}}_{3}$ association step (10) and the corresponding degradation steps (11) are replaced by one lumped step as well:

(16) $${\mathrm{miRNA}}_{2}+{\mathrm{miRNA}}_{3}\to Ø.$$

In this framework, the intermediates, pre-miRNA${}_{2}$ and pre-miRNA${}_{3}$; and complexes, ${\mathrm{mRNA}}_{1}\cdot {\mathrm{miRNA}}_{2}$ and ${\mathrm{premiRNA}}_{2}\cdot {\mathrm{miRNA}}_{3}$, are not described explicitly, and accordingly, the degradation steps of these species ((8) and in (11)) can be excluded from the math. This procedure is intuitively clear. Mathematically, it is a matter of introduction of the effective rate constants (see, e.g., Section 4.2 in [6]).

### 2.3. Mean-Field Kinetic Equations

The coarse-grained scheme introduced above contains cellular mRNA${}_{1}$, P${}_{1}$, and miRNA${}_{2}$; viral miRNA${}_{3}$; and G. The corresponding populations are designated as $n_{1}$, $n_{p}$, $n_{2}$, $n_{3}$, and $n_{g}$. The conventional mean-field kinetic equations for the former four variables are as follows:

(17) $$\begin{array}{c} dn_{1}/dt=w_{1}-k_{1}n_{1}-\kappa_{12}n_{1}n_{2},\\ dn_{p}/dt=\kappa_{p}n_{1}-k_{p}n_{p},\\ dn_{2}/dt=w_{2}-k_{2}n_{2}-\kappa_{12}n_{1}n_{2}-\kappa_{23}n_{2}n_{3},\\ dn_{3}/dt=\kappa_{3}n_{g}-k_{3}n_{3}-\kappa_{23}n_{2}n_{3}, \end{array}$$

where $w_{1}$ and $w_{2}$ are the rates of formation of mRNA${}_{1}$ and miRNA${}_{2}$; $\kappa_{p}$ is the rate constant of the P${}_{1}$ formation; $\kappa_{3}$ is the rate constant of the miRNA${}_{3}$ formation; $\kappa_{12}$ and $\kappa_{23}$ are the association rate constants; and $k_{1}$, $k_{p}$, $k_{2}$, and $k_{3}$ are the degradation rate constants.

According to (17), the P${}_{1}$ population does not influence other populations, and under steady-state conditions this population is just proportional to the mRNA${}_{1}$ population. Under such conditions, it can in principle be excluded from the analysis. I keep P${}_{1}$ for possible extensions of the model. The key argument in favor of P${}_{1}$ is that cellular and viral proteins are widely considered to be main regulators of virus fitness [22,41]. For example, one can assume that P${}_{1}$ regulates the G growth and describe this effect formally by using, e.g., the Hill expression for $\kappa_{g}$ (see below) or in more detail (provided the corresponding information is available).

In addition to (17), I need the equation describing the G replication. The initial phase of this process is expected to be close to exponential. Later on, the exponential growth of the G population is terminated (due to regulation of replication and/or exhaustion of cellular resources), and then this population may diminish (e.g., in the case of cell death). These features follow from numerous experiments with various virus-cell assays (for the types of assays, see [12]) and also from available kinetic models (see, e.g., [21,28] and references therein). In particular, one of the consequences of the exponential growth is that in the corresponding kinetics (e.g., those measured in assays), the logarithm of concentration is customarily shown as a function of time. Phenomenologically, the kinetics of this type can be described as

(18) $$dn_{g}/dt=\kappa_{g}\left(1-n_{g}/n_{*}\right)n_{g},$$

where $\kappa_{g}$ is the replication rate constant and $n_{*}$ is the maximum G population. This equation predicts the exponential growth of the G population at $n_{g}\ll n_{*}$ with subsequent saturation $n_{g}\to n_{*}$. Formally, the two terms, $\kappa_{g}n_{g}$ and $\kappa_{g}n_{g}^{2}/n_{*}$, can be assumed to correspond to forward and backward reactions. Alternatively, the growth can be assumed to be limited by unspecified feedback, and $\kappa_{g}\left(1-n_{g}/n_{*}\right)$ can considered to represent the effective $n_{g}$-dependent replication rate constant. In the mean-field approximation, the equation is applicable in both cases.

Regarding the initial exponential growth predicted by (18), one can for analogy refer to frequently-observed exponential growth of bacteria (briefly reviewed in [42]). In the latter case, the deviations from the exponential growth are sometimes observed as well (briefly reviewed in [43]). Compared to bacterial or cellular DNA, the viral genomes are, however, relatively short, and by analogy with the formation of cellular mRNA and proteins (in the absence of regulation), one can expect that the initial first-order law in (18) is fairly accurate.

Concerning (18), I can add that cellular mRNAs, proteins, and miRNAs, and viral miRNAs, can influence the viral-genome growth due to feedback. In human herpesvirus 6A, for example, virus-encoded miR-aU14 selectively inhibits the processing of multiple miR-30 family members, subsequent activation of the miR-30-p53-DRP1 axis triggers a profound disruption of mitochondrial architecture, and this impairs induction of type I interferons and is necessary for both productive infection and virus reactivation [34]. Although the full-scale analysis of these steps is obviously very interesting, its realization is challenging. One of the already mentioned options is to introduce formal feedback of the Hill type [27,40]. For example, one can admit that the replication rate is positively or negatively regulated by P${}_{1}$ and represent $\kappa_{g}$ in Equation (18) for $n_{g}$, respectively, as

$$\kappa_{g}=\kappa_{g}^{\circ }+\kappa_{g}^{*}\frac{n_{g}^{m}}{K^{m}+n_{p}^{m}}\;\;\mathrm{or}\;\;\kappa_{g}=\kappa_{g}^{\circ }+\kappa_{g}^{*}\frac{K^{m}}{K^{m}+n_{p}^{m}},$$

where $\kappa_{g}^{\circ }$, $\kappa_{g}^{*}$, *K*, and *m* are the Hill parameters. To solve Equation (18), $n_{p}$ should be expressed via $n_{g}$. It can be done by employing Equations (17) above or (19) below. This approach will, however, not really extend the understanding of the mechanistic details. For this reason, I focus on the interplay of mRNA${}_{1}$, P${}_{1}$, miRNA${}_{2}$, and miRNA${}_{3}$ and do not take the effect of these species on the G growth into account—i.e., Equation (18) describing the G replication is considered to be not coupled to Equations (17).

As already noticed, the G-replication timescale, $1/\kappa_{g}$ (Equation (18)), is longer than the timescales characterizing other steps (in (17)), and accordingly, the latter steps can be described in the steady-state approximation by setting $dn_{1}/dt=dn_{p}/dt=dn_{2}/dt=dn_{3}/dt=0$. In this approximation, Equations (17) can be rewritten as

(19) $$\begin{array}{c} n_{1}=w_{1}/\left(k_{1}+\kappa_{12}n_{2}\right),\\ n_{2}=w_{2}/\left(k_{2}+\kappa_{12}n_{1}+\kappa_{23}n_{3}\right),\\ n_{3}=\kappa_{3}n_{g}/\left(k_{3}+\kappa_{23}n_{2}\right),\\ n_{p}=\kappa_{p}n_{1}/k_{p}. \end{array}$$

In these equations for $n_{1}$, $n_{p}$, $n_{2}$, and $n_{3}$, $n_{g}$ can be considered to be a time-dependent governing parameter determined by (18) as

(20) $$n_{g}=\frac{n_{*}\exp\left(\kappa_{g}t\right)}{n_{*}-1+\exp\left(\kappa_{g}t\right)}.$$

This expression shows explicitly $n_{g}$ as a function of $\kappa_{g}t$. For any given $n_{g}$, the other populations, $n_{1}$, $n_{p}$, $n_{2}$, and $n_{3}$, are determined by (19). Thus, $n_{1}$, $n_{p}$, $n_{2}$, and $n_{3}$ can be calculated as a function of $\kappa_{g}t$ as well.

### 2.4. Stochasticity

Stochasticity arising in gene expression from fluctuations in transcription and translation has attracted interest for many years because of its implications for cellular regulation and non-genetic individuality (reviewed in [2,3]; see also recent articles [7,10] and references therein). Compared to conventional chemical systems, the role of fluctuations is here more appreciable because many mRNAs, ncRNAs, and proteins are present in low numbers per cell. During virus replication in cells, the numbers of viral genome, mRNA, ncRNA, and protein copies are often low as well, especially in the beginning. Although the corresponding fluctuations have already been shown theoretically (see, e.g., [15,21,24,25]), the full-scale understanding of their role in real systems is still limited.

The mean-field equations presented above do not take fluctuations into account. To illustrate the scale of fluctuations, one can simulate the steps under consideration by using the MC technique. To simplify simulations, one can notice that the G population is typically smaller than the mRNA${}_{1}$ and miRNA${}_{2}$ populations and often smaller than the miRNA${}_{3}$ population. This means that the generation of an additional G copy results in a rapid, appreciable increase or decrease in numbers of copies of other species at the mean-field level. Under such conditions, the fluctuations of the mRNA${}_{1}$, P${}_{1}$, miRNA${}_{2}$, and miRNA${}_{3}$ populations are related primarily to those of the G population, and accordingly, the MC technique, or more specifically, the standard Gillespie algorithm, can be used in order to describe only this population, i.e., $n_{g}$ (Equation (18)), whereas the populations of other species can be tracked at any given $n_{g}$ at the mean-field level by employing (19). The realization of the corresponding MC simulations depends slightly on the interpretation of Equation (18). To be specific, I consider that the G growth is limited by unspecified feedback so that $\kappa_{g}\left(1-n_{g}/n_{*}\right)$ represents the effective replication rate constant. In this framework, the time interval corresponding the generation of an additional G copy is given by $\Delta t=\ln\left(q\right)/[\kappa_{g}\left(1-n_{g}/n_{*}\right)n_{g}]$, where 0<q≤1 is a random number, and $\kappa_{g}\left(1-n_{g}/n_{*}\right)n_{g}$ is the replication rate (in (18)).

## 3. Results and Discussion

### 3.1. Mean-Field Kinetics

The coarse-grained model introduced above is focused on five key species, including cellular mRNA${}_{1}$, P${}_{1}$, and miRNA${}_{2}$; viral miRNA${}_{3}$; and G. Equations (19) and (20) for the corresponding populations, $n_{1}$, $n_{p}$, $n_{2}$, $n_{3}$, and $n_{g}$, contain many kinetic parameters. In principle, the model can be used in order to describe real kinetics of infection of individual cells, and ideally, the parameters can/should be specified by using independent experiments. In practice, with the current state of the art, this is impossible because the available experimental data are far from sufficient. Under such circumstances, my goal is less ambitious. Specifically, I focus on the typical shape of the kinetics under consideration with biologically reasonable populations of various species. Following this line, I show below the populations of cellular mRNA${}_{1}$ and miRNA${}_{2}$, $n_{1}$ and $n_{2}$; viral miRNA${}_{3}$, $n_{3}$; and G, $n_{g}$. The P${}_{1}$ population, $n_{p}=\kappa_{p}n_{1}/k_{p}$ (in (19)), is not shown, because this population is just proportional to the mRNA${}_{1}$ population.

To keep the presentation of the result transparent and compact, I set the rate constants of degradation of mRNA${}_{1}$, miRNA${}_{2}$, and miRNA${}_{3}$ to be equal (to *k*), i.e., $k_{1}=k_{p}=k_{2}=k_{3}\equiv k$. In addition, I took into account that, in fact, the solution of Equations (19) depends only on the ratios of various rate constants, and accordingly, one can operate by using these ratios. The corresponding dimensionless values should be chosen to have biologically reasonable populations of mRNA${}_{1}$, miRNA${}_{2}$, and miRNA${}_{3}$. In human cells, the populations of these species are well known to be in a wide range, roughly from 10 to 10${}^{4}$ per cell (see, e.g., [6]). To be in this range, I used somewhat arbitrarily the following set of the parameters: $w_{1}/k=200$, $w_{2}/k=400$, $\kappa_{12}/k=0.01$, $\kappa_{23}/k=0.01$, and $\kappa_{3}/k=10$.

**Figure 1:**
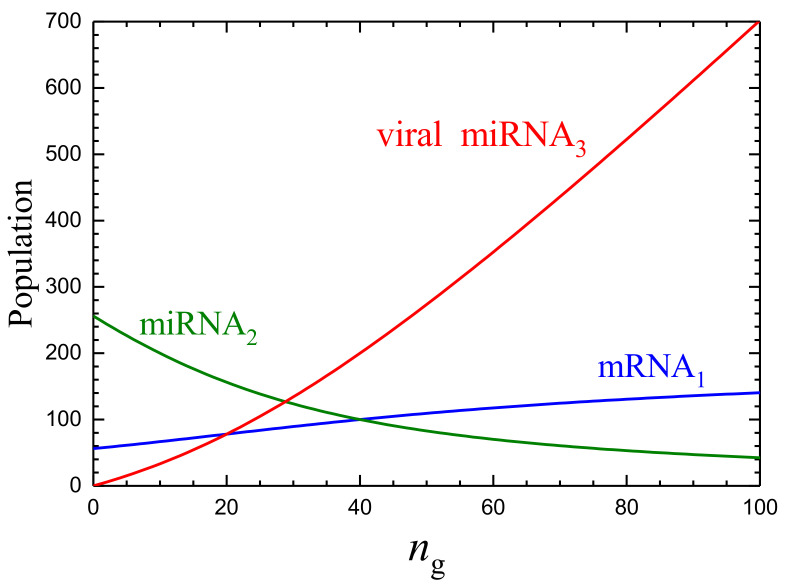
mRNA${}_{1}$, miRNA${}_{2}$, and miRNA${}_{3}$ populations as a function of the G population according to (19) with $w_{1}/k=200$, $w_{2}/k=400$, $\kappa_{12}/k=\kappa_{23}/k=0.01$, and $\kappa_{3}/k=10$. The model contains also the P${}_{1}$ population. The latter population (not shown here and below in other figures) is just proportional to the mRNA${}_{1}$ population.

**Figure 2:**
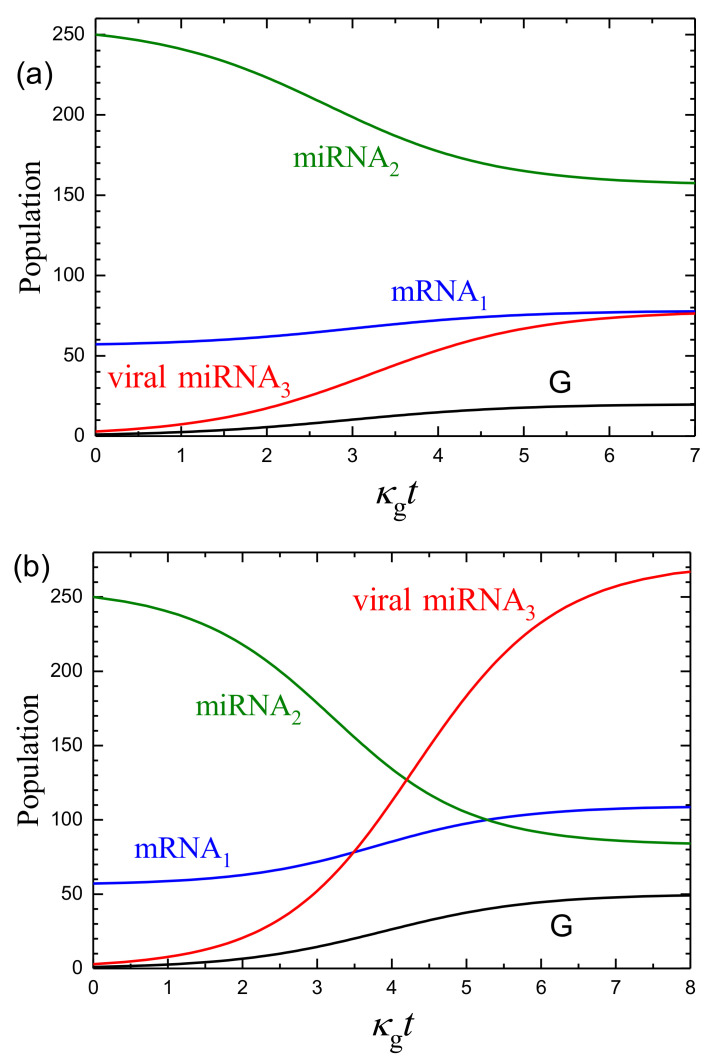

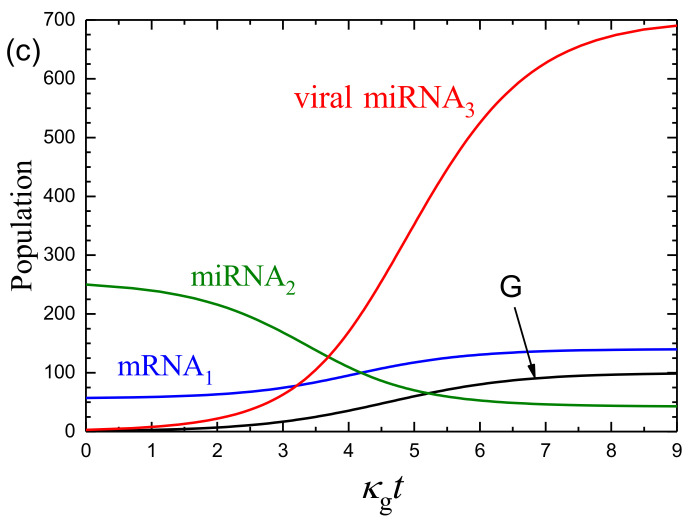
mRNA${}_{1}$, miRNA${}_{2}$, miRNA${}_{3}$, and G and populations as a function of $\kappa_{g}t$ according to Equations (19) and (20) with $n_{g}\left(0\right)=1$ for $n_{*}=20$ (**a**), 50 (**b**), and 100 (**c**) and the other parameters ($w_{1}/k=200$, $w_{2}/k=400$, $\kappa_{12}/k=\kappa_{23}/k=0.01$, and $\kappa_{3}/k=10$) as in Figure 1. In this model, the infection of a cell is possible by one virion [$n_{g}\left(0\right)=1$], in agreement with the so-called independent action hypothesis [40]. In the absence of virus ($n_{g}=0$ and $n_{3}=0$), the mRNA${}_{1}$ and miRNA${}_{2}$ population are $n_{1}=56$ and $n_{2}=256$ (Figure 1).

The steady-state dependence of the mRNA${}_{1}$, miRNA${}_{2}$, and miRNA${}_{3}$ populations, $n_{1}$, $n_{2}$, and $n_{3}$, on the G population, $n_{g}$, predicted by (19), with the chosen values of the kinetic parameters is shown in Figure 1. The temporal mean-field kinetics under consideration; or more specifically, the mRNA${}_{1}$, miRNA${}_{2}$, miRNA${}_{3}$, and G populations calculated according to Equations (19) and (20) as a function of $\kappa_{g}t$ are exhibited in Figure 2.

To interpret the kinetics shown in Figure 1 and Figure 2, one can notice that in the absence of the mRNA${}_{1}$-miRNA${}_{2}$ and miRNA${}_{2}$-miRNA${}_{3}$ interactions, the model with the chosen parameters predicts that the mRNA${}_{1}$ and miRNA${}_{2}$ populations should be $n_{1}=w_{1}/k=200$ and $n_{2}=w_{2}/k=400$. These values are typical for abundant mRNAs and miRNAs. In the absence of the miRNA${}_{2}$–miRNA${}_{3}$ interaction, the mRNA${}_{1}$–miRNA${}_{2}$ interaction results in a decrease in the mRNA${}_{1}$ and miRNA${}_{2}$ populations down to $n_{1}=56$ and $n_{2}=256$ (this case corresponds to $n_{g}=0$ in Figure 1). The scale of this decrease is typical for the mRNA–miRNA interaction. In the presence the miRNA${}_{2}$–miRNA${}_{3}$ interaction, the effect of miRNA${}_{3}$ on the mRNA${}_{1}$ and miRNA${}_{2}$ populations is first nearly negligible as long as the miRNA${}_{3}$ population is low. With increasing G and miRNA${}_{3}$ populations, the effect of miRNA${}_{3}$ on the miRNA${}_{2}$ population becomes larger, and the latter population drops, and accordingly, the suppression of the mRNA${}_{1}$ population becomes weaker. Thus, the mRNA${}_{1}$ population grows. If the miRNA${}_{3}$ population eventually reaches an appreciable value (as, e.g., in Figure 2c), the mRNA${}_{1}$ population increases up to 140, i.e., by a factor of 2.

**Figure 3:**
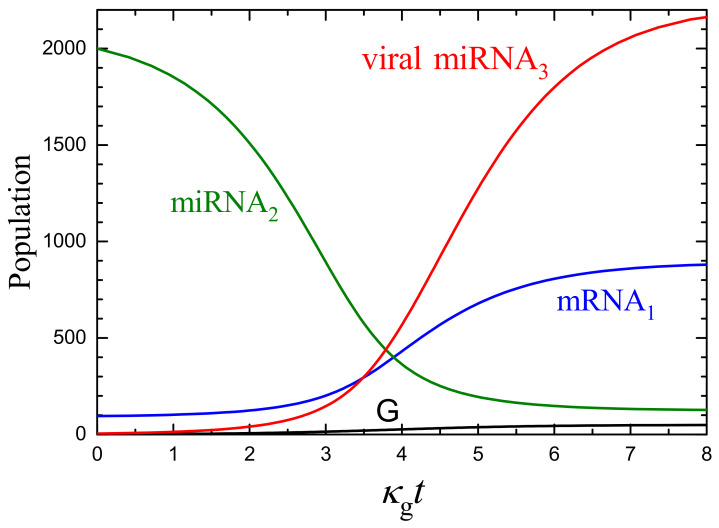
As Figure 2 for $w_{1}/k=2000$, $w_{2}/k=4000$, $\kappa_{12}/k=\kappa_{23}/k=0.01$, $\kappa_{3}/k=100$, and $n_{*}=50$. Compared to the parameters used to construct Figure 2, $w_{1}/k$, $w_{2}/k$, and $\kappa_{3}/k$ are here increased by one order of magnitude, whereas $n_{*}=50$ is as in Figure 2b. In the absence of virus ($n_{g}=0$ and $n_{3}=0$), the mRNA${}_{1}$ and miRNA${}_{2}$ population are $n_{1}=91$ and $n_{2}=2091$.

To illustrate the effect of the variation of parameters on the kinetics, it is instructive to increase the rate constants of the formation of mRNA${}_{1}$, miRNA${}_{2}$, and miRNA${}_{3}$ by one order of magnitude up to $w_{1}/k=2000$, $w_{2}/k=4000$, and $\kappa_{3}/k=100$ and to keep other parameters the same as above ($\kappa_{12}/k=0.01$ and $\kappa_{23}/k=0.01$). In this case, the initial miRNA${}_{2}$ population becomes larger, roughly by one order of magnitude (cf. Figure 2b and Figure 3). The initial mRNA${}_{1}$ population increases only by a factor of 2 because the increase in $w_{1}/k$ is compensated by the increase in the initial miRNA${}_{2}$ population. Globally, the kinetics are qualitatively the same. Quantitatively, the final relative increase in the mRNA${}_{1}$ population and decrease in the miRNA${}_{2}$ population (compared to the initial values) are more appreciable.

For arbitrary parameters, the relative roles of various processes can be clarified by analogy under the steady-state conditions (by using (19), first in the case before infection (with $n_{g}=0$) and then for an appreciable $n_{g}$, e.g., 100, or under transient conditions (by employing (19) and (20)).

### 3.2. Monte Carlo Simulations

**Figure 4:**
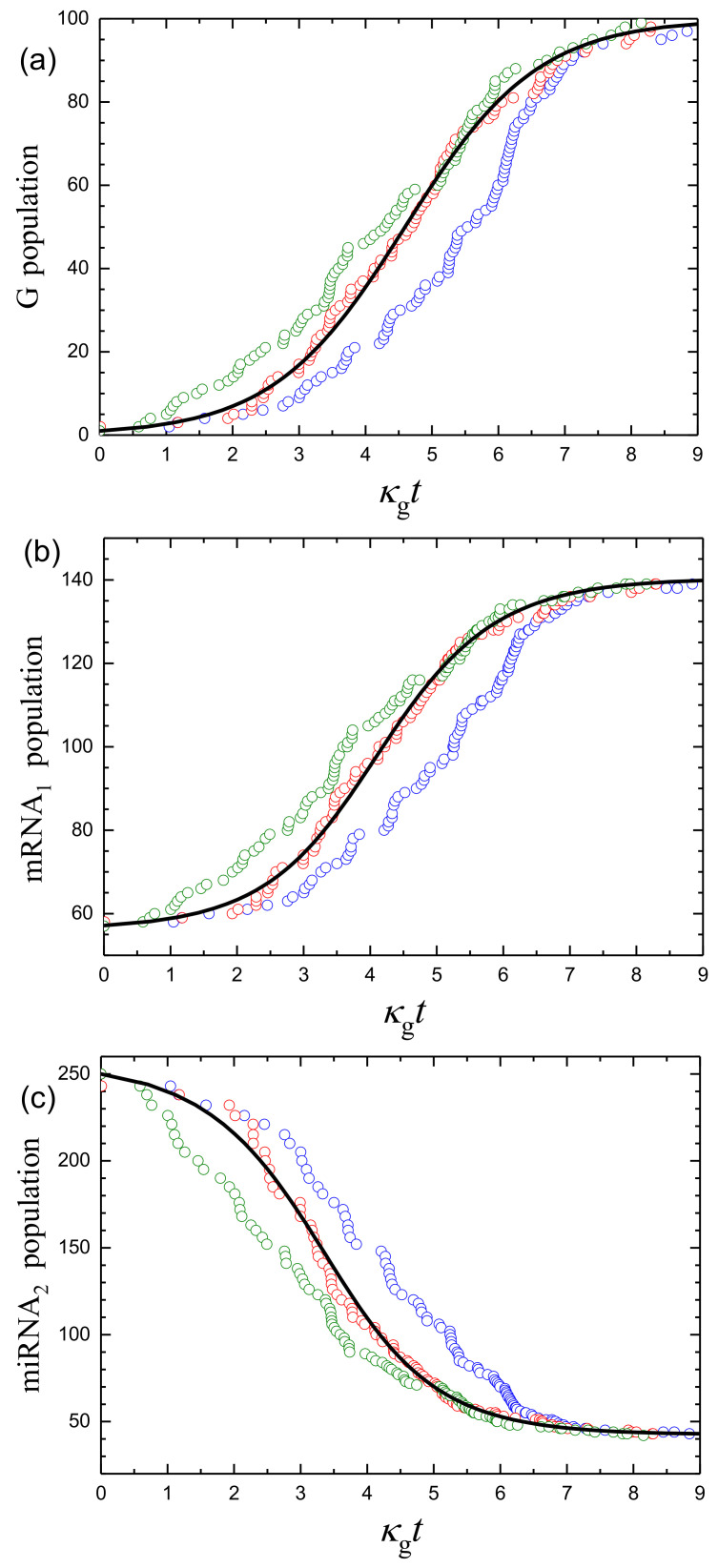

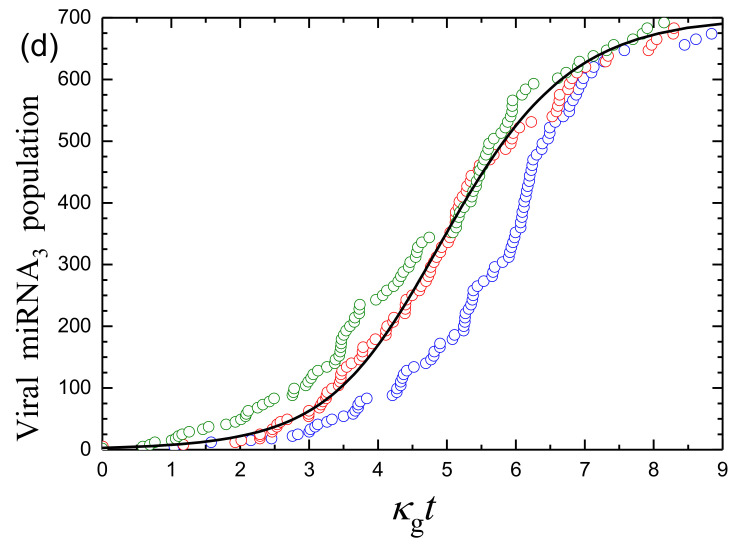
(**a**) G, (**b**) mRNA${}_{1}$, (**c**) miRNA${}_{2}$, and (**d**) miRNA${}_{3}$ populations as a function of $\kappa_{g}t$ for $n_{g}\left(0\right)=1$. The mean-field kinetics calculated according to Equations (19) and (20) are shown by solid lines. Open circles exhibit three runs of the corresponding MC kinetics. The parameters are as in the case of Figure 2c.

**Figure 5:**
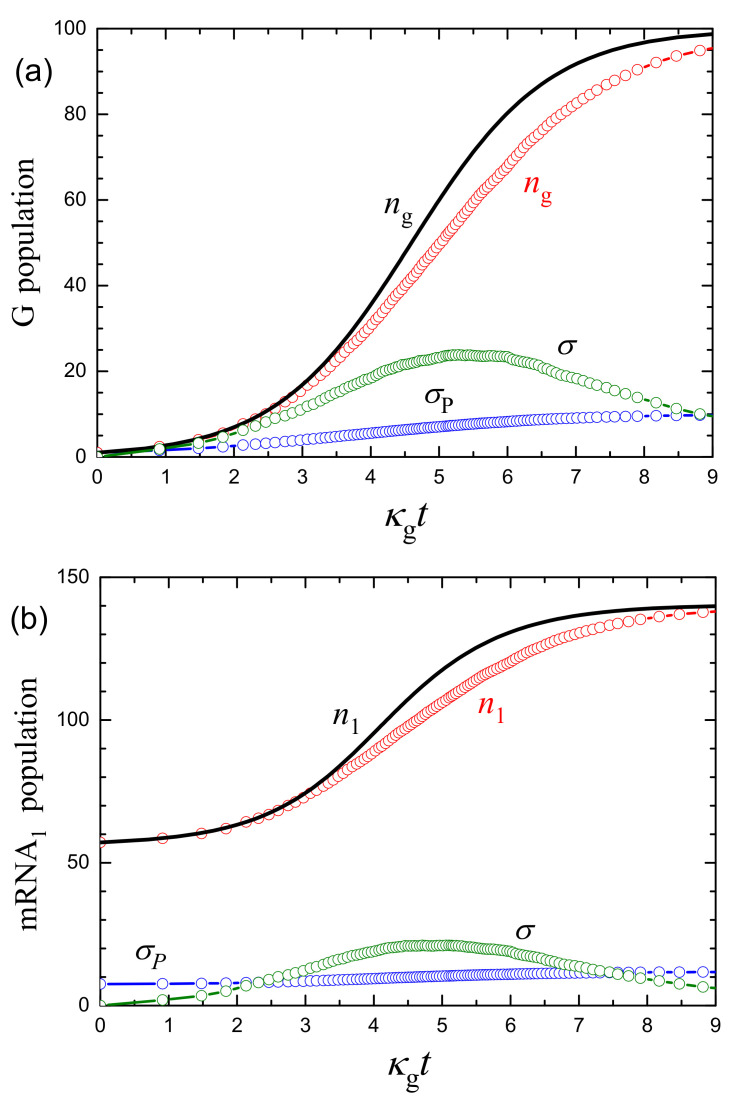

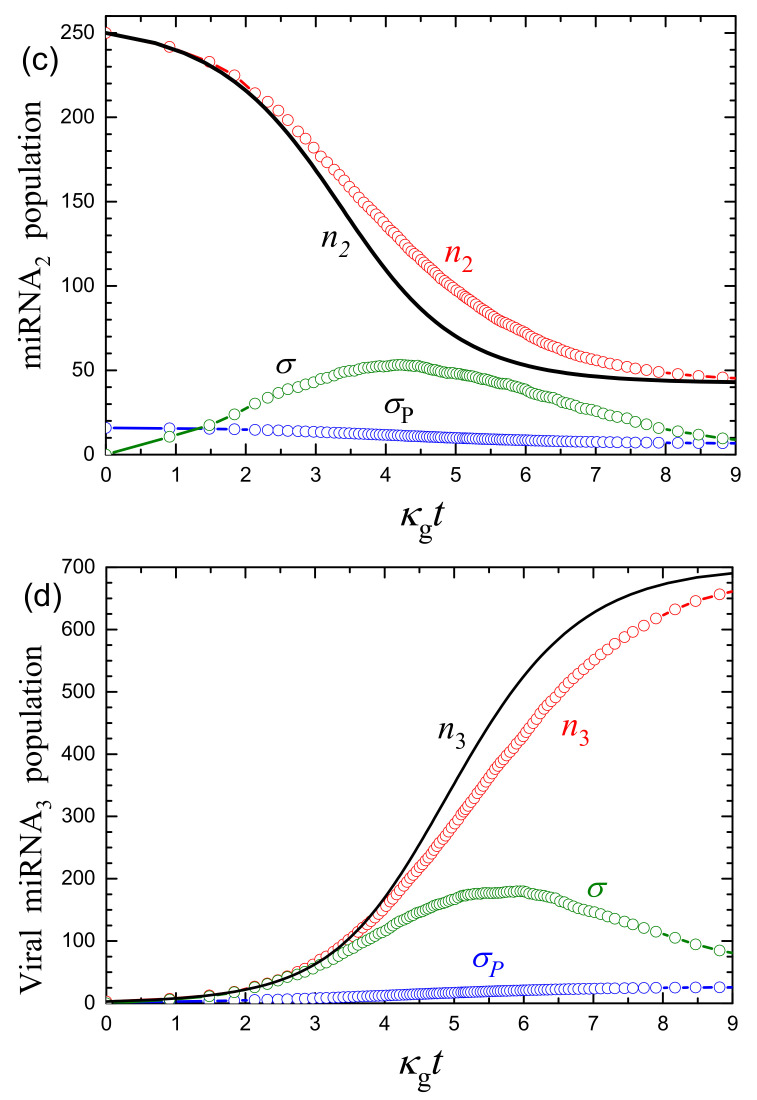
(**a**) G, (**b**) mRNA${}_{1}$, (**c**) miRNA${}_{2}$, and (**d**) miRNA${}_{3}$ kinetics as a function of $\kappa_{g}t$ for $n_{g}\left(0\right)=1$. The mean-field populations (Equations (19) and (20)) are shown by solid lines. The averaged MC populations, standard deviations (Equation (21)), and Poissonian deviations (Equation (23)) shown by open circles were calculated by using 100 MC runs (as those exhibited in Figure 4).

Typical temporal MC kinetics (Figure 4) exhibit appreciable fluctuations. Such kinetics can be characterized by calculating average populations, 〈n〉, and the corresponding standard deviations (Figure 5),

(21) $$\sigma ={\langle {\left(\Delta n\right)}^{2}\rangle }^{1/2}.$$

To interpret the results presented (Figure 5), I recall that in the context of gene expression in cells, each standard deviation (e.g., that corresponding to protein) is usually represented as a sum of three counterparts (see, e.g., [7] and note that the terminology I use is slightly different),

(22) $$\sigma =\sigma_{\mathrm{in}}+\sigma_{\mathrm{ex}}+\sigma_{\mathrm{div}},$$

where $\sigma_{\mathrm{in}}$ is the intrinsic part (under steady-state conditions, this term is associated with Poisson noise); $\sigma_{\mathrm{ex}}$ is the extrinsic part related to regulation, or in other words, to the upstream noise (e.g., the fluctuations in mRNA copy number in the case of protein); and $\sigma_{\mathrm{div}}$ is the part related to partitioning noise due to the cell division. The Poisson distribution is well known to yield

(23) $$\sigma_{P}={\langle n\rangle }^{1/2},$$

and under steady-state conditions with detailed balance, one is expected to have $\sigma =\sigma_{\mathrm{in}}=\sigma_{P}$. Using 〈n〉, $\sigma_{P}$ can formally be calculated under transient conditions as well (Equation (23)). In the latter case, σ can deviate from $\sigma_{P}$.

In the model under consideration, the growth and division of cells are ignored, and accordingly, $\sigma_{\mathrm{div}}=0$ for all the species. The viral genome replication (step (12)) is considered to be independent of mRNA${}_{1}$, P, miRNA${}_{2}$, and miRNA${}_{3}$—i.e., there is no regulation ($\sigma_{\mathrm{ex}}=0$)—and accordingly, the fluctuations are intrinsic, i.e., $\sigma =\sigma_{\mathrm{in}}$. In contrast, mRNA${}_{1}$, P, miRNA${}_{2}$, and miRNA${}_{3}$ are described at the mean-field level at each G copy number; i.e., the fluctuations of these species are related to those of the G population, and this means that $\sigma_{\mathrm{in}}=0$ and $\sigma =\sigma_{\mathrm{ex}}$.

The simulations show that for G replication, σ is close to $\sigma_{P}$ in the beginning and end of the kinetics, and larger than $\sigma_{P}$ by a factor of three in the medium, where the G growth is fastest (Figure 5a). The miRNA${}_{3}$ population is directly dependent on the G population, and here σ is much larger than $\sigma_{P}$ (Figure 5d). The mRNA${}_{1}$ and miRNA${}_{2}$ population are indirectly dependent on the G population, and for them σ is smaller than $\sigma_{P}$ in the beginning, smaller than or comparable to $\sigma_{P}$ in the end, and appreciably larger than $\sigma_{P}$ in the medium of the kinetics (Figure 5b,c). Thus, the scales of the maximal fluctuations of the mRNA${}_{1}$, miRNA${}_{2}$, and miRNA${}_{3}$ populations are rather appreciable compared to $\sigma_{P}$. As already noticed, the reason for this feature is that the fluctuations of the populations of these species are related directly or indirectly to those of the G population. The latter population is relatively small, the fluctuations of this population with respect to its average value (the corresponding measure is σ/〈n〉) are relatively large, and accordingly, the fluctuations of other populations are large as well.

## 4. Conclusions

Viral miRNAs can influence the kinetics of gene expression during infection of cells. This is an interesting example of transient kinetics of gene expression. In this study, I have presented a generic kinetic model and results of mean-field calculations and MC simulations illustrating the specifics of the interplay of viral miRNAs and cellular mRNAs, proteins, and miRNAs. The predicted type and scale of the effect of viral miRNA on cellular species are in qualitative agreement with the experiments reported for herpesvirus [34]. Quantitative theoretical analysis of the experiments is now hardly possible, because, as it often happens, the reported experimental data viewed from the perspective of the theory are incomplete.

Finally, I can note that the model presented can be extended in various directions. (i) For example, the model implies that the protein formed after translation of cellular mRNA does not regulate the formation of mRNA and other species. In reality, this regulation can take place, and it can result in more complex kinetics, including, e.g., bistability or oscillations [6]. (ii) The regulation of the replication of viral genomes can be taken into account (in more detail compared to [27,40]). (iii) Another extension can be aimed at more detailed analysis of what may happen at high intracellular population of viral genomes in the limit when the genome kinetic is not exponential (in the spirit of Ref. [21]). (iv) The cell cycle (or death) can be introduced into the treatment as well (in the spirit of references [44,45,46]). (v) Stochastic effects can be analyzed in more detail. (vi) All these extensions ((i)–(v)) can be done at the level of a few species. The specifics of miRNAs is that each such species can associate with various mRNAs [47], and the corresponding genetic networks can include multiple steps (up to one hundred). Such large networks can be analyzed as well.

## Data Availability

The data presented in this study are available in the article.
